# Global burden and projection of colorectal cancer attributable to low whole-grain diets: an analysis of GBD 2021 data with Bayesian age-period-cohort modeling

**DOI:** 10.3389/fonc.2025.1572053

**Published:** 2025-07-16

**Authors:** Hongmei Tian, Xin Xie, Xiaoqing Li, Yueshan Pang, Jiebin Xie

**Affiliations:** ^1^ Department of Nursing, Affiliated Hospital of North Sichuan Medical College, Nanchong, Sichuan, China; ^2^ Department of Gastrointestinal Surgery, Affiliated Hospital of North Sichuan Medical College, Nanchong, Sichuan, China; ^3^ Department of Critical Care Medicine, Affiliated Hospital of North Sichuan Medical College, Nanchong, Sichuan, China; ^4^ Department of General Practice, Beijing Anzhen Nanchong Hospital of Capital Medical University and Nanchong Central Hospital, Sichuan, China

**Keywords:** whole-grain diets, GBD, colon cancer, rectal cancer, BAPC projection analysis, CRC

## Abstract

**Background:**

A diet low in whole grains may be a significant risk factor for colorectal cancer (CRC). Therefore, analyzing the latest global burden of disease (GBD) data to understand the burden of CRC attributable to low whole-grain diets is crucial for informing public health policies aimed at reducing CRC-related burdens.

**Methods:**

This study utilized data from the GBD 2021 to analyze global trends in mortality and disability-adjusted life years (DALYs) attributable to low whole-grain diets from 1990–2021. A Bayesian age-period-cohort (BAPC) model was employed to project the future burden of CRC attributable to low whole-grain diets up to 2050.

**Results:**

In 2021, the global number of deaths attributable to low whole-grain diets was 186,256.80, representing an 82.94% increase from 1990. The number of global DALYs attributable to low whole-grain diets in 2021 was 4,327,218.86, a 70.30% increase from 1990. The burden of CRC in males attributable to low whole-grain diets was greater than that in females aged 50–74 years. Nationally, Monaco recorded the highest mortality rate (13.23/100,000), China had peak deaths/DALYs. Projections to 2050, the global number of deaths attributable to low whole-grain diets will continue to rise, reaching 199,565.06.

**Conclusions:**

Low whole-grain diets pose a significant threat to public health, contributing to an increasing burden of CRC. To reduce the burden of CRC attributable to low whole-grain diets, enhancing public education and awareness of whole-grain diets, implementing policies to promote the consumption of whole grains, and conducting early screening among high-risk populations are recommended.

## 1 Introduction

Colorectal cancer (CRC) ranks among the top three malignancies globally and is the second primary cause of cancer death worldwide (1). Although preliminary screening and diagnosis have reduced CRC mortality in recent years, the death rate remains high (2). In the United States, an estimated 153,020 new CRC cases and 52,550 CRC-related deaths were attributed to the disease in 2023 (3). Unhealthy dietary habits are a major risk factor for the increasing global prevalence of CRC, particularly among younger populations (4, 5). In 2019, dietary risk factors accounted for 365,752 CRC-related deaths, accounting for approximately one-third of deaths related to CRC (4). Therefore, updating the burden of CRC attributable to dietary factors via the latest GBD data is essential for increasing public awareness and guiding health policy decisions to reduce CRC-related burdens.

The World Health Organization and global dietary guidelines recognize whole grains as crucial components of a healthy diet. A diet low in whole grains is associated with various chronic diseases (6, 7) and is a significant dietary risk factor for CRC (4, 8). In the United States, older men with high whole-grain intake have a 43% lower risk of rectal cancer than do those with low intake (9). Globally, the burden of CRC attributable to low whole-grain diets is substantial, with 171487 deaths attributed to low whole-grain diets in 2019 (10). Despite the proven health benefits of whole grains, economic development, changing lifestyles, and increased consumption of processed foods have led to a decline in whole-grain intake (11, 14). Consequently, low whole-grain diets have become a global public health concern, particularly in relation to CRC.

The Global Burden of Disease (GBD) initiative meticulously compiles and assesses epidemiological data concerning numerous diseases and injuries from diverse sources. This study leverages the latest GBD data to analyze trends in mortality and DALYs attributable to low whole-grain diets from 1990–2021, stratified by sex, age, and SDI. The findings aim to inform public health policies and interventions to reduce the burden of CRC attributable to low whole-grain diets.

## 2 Methods

### 2.1 Data acquisition and sources

The GBD 2021 dataset, which evaluates the effects of 371 diseases and injuries along with 88 risk factors across 204 countries and territories from 1990–2021, was used to gather data for this study (12). Our focus was on the data regarding mortality and DALYs of CRC attributable to low whole-grain diets. The dataset was retrieved and downloaded via the Global Health Data Exchange platform (https://vizhub.healthdata.org/gbd-results). This study was purely analytical, with no personal identifiable information processed. Data on the sociodemographic index (SDI) were also collected to evaluate the influence of socioeconomic factors on disease burden.

### 2.2 Temporal trend analysis

This study utilized joinpoint regression analysis to assess the longitudinal trends in deaths and DALYs due to low whole-grain diets between 1990 and 2021. The “Segment” and “broom” R packages were used to identify significant shifts in trends over time. The estimated annual percentage change (EAPC) was calculated via JD_GBDR (V2.30, Jingding Medical Technology Co., Ltd.), and the statistical significance of these trends was assessed via 95% confidence intervals (CIs).

### 2.3 Population analysis

Demographic analyses were conducted to investigate the prevalence of CRC attributable to low whole-grain diets, stratified by age and sex. The dataset was divided into three age groups (15–49 years, 50–74 years, and 75+ years) for both males and females. Data processing and visualization were performed via the R programming language and the “ggplot2” package.

### 2.4 SDI analysis

The relationship between the SDI and the burden of CRC attributable to low whole-grain diets was examined by calculating SDI-specific disease rates. SDI categories were used to compare disease burdens across different levels of socioeconomic development. The “dplyr” and “ggplot2” packages in R were used for data manipulation and visualization.

### 2.5 Global and regional burden analysis

Global cartography and regional comparative studies have been conducted to examine the worldwide distribution and regional variations in the deaths, DALYs and EAPCs of CRC attributable to low whole-grain diets. The data were consolidated according to the geographical divisions established by the GBD study. R (version 4.2.3) and the “rnaturalearthdata” package were used to create visual representations of the geographical spread of disease burdens.

### 2.6 Bayesian age–period–cohort model for forecasting

A BAPC model was applied to predict the future burden of CRC attributable to low whole-grain diets. The model, which uses the ‘INLA’ and ‘BAPC’ packages in R for implementation, forecasts CRC mortality attributable to low whole-grain diets through 2050, considering the effects of age, period, and cohort.

### 2.7 Statistical analysis

The statistical calculations and graphical outputs were generated via R software (version 4.2.3) and JD_GBDR (V2.24, Jingding Medical Technology Co., Ltd.). Descriptive statistics were generated for each principal variable, with the results presented as the mean values accompanied by 95% CIs or uncertainty intervals (UIs). In the trend analyses, statistical significance was attributed to p values less than 0.05.

## 3 Results

### 3.1 Global burden of CRC attributable to low whole-grain diets

Globally, the disease burden of CRC attributable to low whole-grain diets has increased significantly. In 1990, the global number of deaths attributable to low whole-grain diets was 101,812.86 (95% UI: 42,588.20–151,170.08). By 2021, the global number of deaths had increased to 186,256.80 (95% UI: 76,126.73–284,803.37). From 1990–2021, global deaths attributable to low whole-grain diets increased significantly by 82.94% (95% UI: 67.76–98.05), accompanied by a rise in the mortality rate from 1.91 to 2.36 per 100,000 people, with an EAPC of 0.64% (95% CI: 0.58–0.69) (**Table 1**). The number of global DALYs related to low whole-grain diets increased by 70.30% (95% UI: 55.66–86.21) from 1990 to 2021, increasing from 2.54 million to 4.33 million years, whereas the percentage per 100,000 people climbed from 47.64 to 54.83 years (**Supplementary Table 1**).

**Table 1:** Deaths of CRC attributable to low whole-grain diets between 1990–2021 at the global and regional level.

Location	1990 (95% UI))		2021 (95% UI)		Case percent change % (95% UI)	Rate EAPC % (95% CI)
	Case	Rate	Case	Rate		
Global	101,812.86 (42,588.20–151,170.08)	1.91 (0.80–2.83)	186256.80 (76126.73–284,803.37)	2.36 (0.96–3.61)	82.94 (67.76–98.05)	0.64 (0.58–0.69)
High SDI	43,042.62 (18,344.92–64,228.50)	4.89 (2.09–7.30)	60,473.36 (25,199.86–92,184.17)	5.53 (2.30–8.43)	40.50 (32.17–46.85)	0.30 (0.26–0.35)
High-middle SDI	31,689.75 (13,145.57–46,872.58)	2.98 (1.24–4.41)	56,721.44 (22,911.24–86,094.51)	4.35 (1.76–6.60)	78.99 (61.29–99.03)	1.18 (1.14–1.22)
Middle SDI	18,434.85 (7,398.07–28,070.12)	1.07 (0.43–1.63)	47,737.84 (19,196.51–73,112.02)	1.95 (0.78–2.99)	158.95 (118.55–202.21)	1.95 (1.86–2.05)
Low-middle SDI	5,747.62 (2,416.57–8,914.23)	0.49 (0.21–0.77)	15,442.23 (6,372.49–22,973.55)	0.80 (0.33–1.20)	168.67 (132.34–221.50)	1.63 (1.57–1.70)
Low SDI	2,751.01 (1,161.20–4,291.23)	0.55 (0.23–0.86)	5,639.91 (2,327.41–8,439.87)	0.50 (0.21–0.76)	105.01 (76.33–177.68)	-0.39 (-0.56–0.21)
Regions						
Andean Latin America	310.96 (132.58–475.22)	0.82 (0.35–1.25)	1,024.08 (391.53–1,604.68)	1.55 (0.59–2.43)	229.33 (165.65–302.96)	2.38 (2.24–2.51)
Australasia	1,009.37 (421.25–1,533.18)	4.98 (2.08–7.56)	1,479.30 (601.99–2,258.81)	4.78 (1.94–7.30)	46.56 (28.28–66.44)	-0.35 (-0.43–0.26)
Caribbean	636.70 (271.92–959.90)	1.80 (0.77–2.72)	1,426.41 (562.20–2,167.07)	3.01 (1.18–4.57)	124.03 (93.75–154.28)	1.80 (1.76–1.85)
Central Asia	907.98 (379.45–1365.31)	1.31 (0.55–1.97)	1,163.26 (480.67–1,783.85)	1.21 (0.50–1.86)	28.12 (12.74–44.17)	0.08 (-0.09–0.25)
Central Europe	5,914.65 (2477.60–8741.12)	4.73 (1.98–6.99)	9,495.24 (3,942.10–14,239.80)	8.24 (3.42–12.35)	60.54 (47.90–73.54)	1.81 (1.71–1.91)
Central Latin America	925.82 (380.44–1365.59)	0.56 (0.23–0.83)	3795.31 (1,513.46–5,782.64)	1.50 (0.60–2.29)	309.94 (266.60–354.17)	3.30 (3.18–3.42)
Central Sub-Saharan Africa	255.98 (108.68–396.93)	0.47 (0.20–0.72)	645.35 (261.03–1088.35)	0.47 (0.19–0.79)	152.11 (92.71–254.39)	-0.02 (-0.22–0.17)
East Asia	22,088.23 (8,770.80–34,016.10)	1.81 (0.72–2.79)	52,282.82 (20,975.52–83,298.33)	3.55 (1.42–5.66)	136.70 (80.46–200.60)	2.17 (2.09–2.26)
Eastern Europe	9,452.69 (3,919.93–14,005.83)	4.17 (1.73–6.18)	12,006.84 (5,039.47–17,765.90)	5.81 (2.44–8.59)	27.02 (16.18–38.41)	0.84 (0.71–0.96)
Eastern Sub-Saharan Africa	1,364.60 (574.84–2131.20)	0.72 (0.30–1.12)	2,773.89 (1,188.77–4,139.98)	0.65 (0.28–0.97)	103.28 (67.32–199.53)	-0.47 (-0.70–0.25)
High-income Asia Pacific	5,843.15 (2,460.66–8,830.50)	3.37 (1.42–5.09)	13,974.22 (5,930.76–21,445.21)	7.54 (3.20–11.56)	139.16 (109.45–158.08)	2.61 (2.54–2.68)
High-income North America	13,194.37 (5,633.05–19,616.74)	4.69 (2.00–6.97)	15,389.81 (6,563.53–23,033.01)	4.16 (1.77–6.22)	16.64 (11.95–21.06)	-0.54 (-0.61–0.46)
North Africa and Middle East	2637.90 (1,069.56–3,947.36)	0.78 (0.32–1.16)	7,009.92 (2,866.51–10,716.52)	1.13 (0.46–1.72)	165.74 (128.48–220.87)	1.40 (1.21–1.59)
Oceania	30.40 (12.27–47.51)	0.46 (0.19–0.73)	70.29 (27.96–106.91)	0.50 (0.20–0.77)	131.22 (92.78–178.13)	0.29 (0.14–0.43)
South Asia	4,280.28 (1859.37–6563.94)	0.39 (0.17–0.60)	11,355.69 (4735.65–17,076.82)	0.61 (0.26–0.92)	165.30 (116.74–232.24)	1.36 (1.21–1.52)
Southeast Asia	3,950.46 (1640.02–6042.76)	0.85 (0.35–1.30)	12,528.26 (5,042.44–18,992.75)	1.79 (0.72–2.72)	217.13 (168.50–272.76)	2.41 (2.39–2.44)
Southern Latin America	1,697.05 (710.22–2524.85)	3.43 (1.43–5.10)	3,019.80 (1,248.03–4,597.84)	4.46 (1.84–6.79)	77.94 (55.02–104.78)	1.07 (0.90–1.25)
Southern Sub-Saharan Africa	347.48 (146.05–542.59)	0.66 (0.28–1.04)	969.29 (408.64–1475.94)	1.21 (0.51–1.84)	178.95 (144.13–221.12)	2.12 (1.88–2.35)
Tropical Latin America	1426.88 (590.48–2142.98)	0.94 (0.39–1.40)	5029.23 (2143.76–7573.20)	2.21 (0.94–3.33)	252.46 (228.54–275.45)	2.86 (2.80–2.93)
Western Europe	24,784.51 (10,511.13–36,960.19)	6.45 (2.73–9.61)	28,984.77 (11,875.46–43,653.13)	6.63 (2.72–9.98)	16.95 (7.42–24.86)	0.05 (0.02–0.09)
Western Sub-Saharan Africa	753.40 (307.40–1151.63)	0.39 (0.16–0.60)	1,833.04 (788.27–2756.76)	0.37 (0.16–0.56)	143.30 (97.87–205.58)	-0.03 (-0.11–0.05)

CRC, colorectal cancer; DALY, disability-adjusted life years; EAPC, estimated annual percentage change; CI, confidence interval;UI, uncertainty interval.

**Figure 1:**
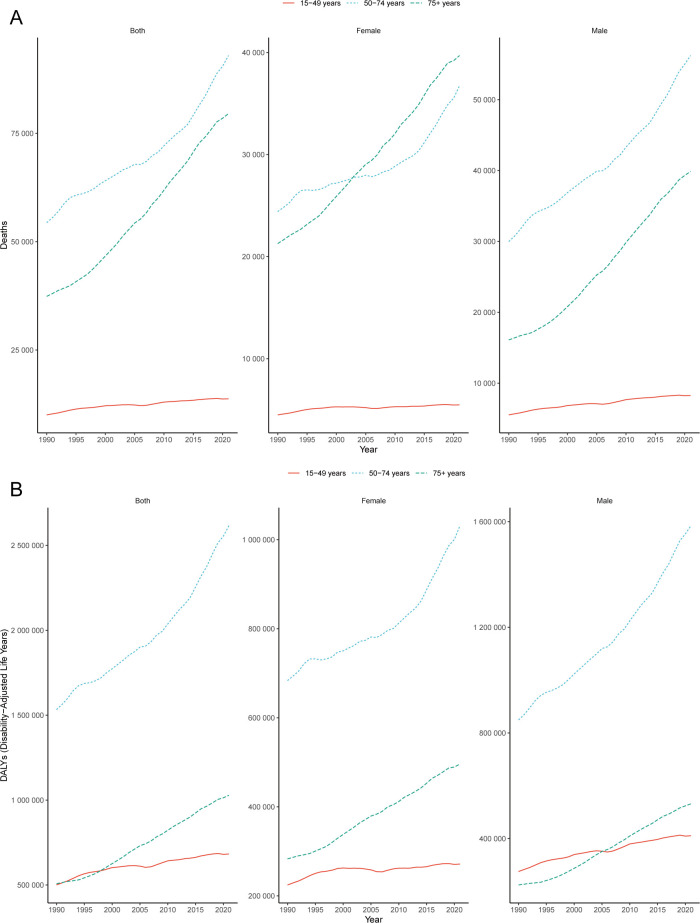
Global burden of CRC. Trends in CRC deaths **(A)** and DALYs **(B)** attributable to low whole-grain diets, 1990–2021. CRC, colorectal cancer; DALY, disability-adjusted life years.

From 1990 to 2021, the greatest increase in mortality among the total population (both sexes combined) occurred in the 50–74 years age group, with a rise of 70.88% (95% UI: 55.65–86.55) (**Figure 1A**). Among females, the most significant increase in mortality was observed in the 75+ years age group, with an increase of 86.55% (95% UI: 68.80–100.76) (**Figure 1A**). In contrast, among males, the greatest increase in mortality was observed in the 50–74 years age group, with an increase of 87.49% (95% UI: 65.06–113.91) (**Figure 1A**). From 1990–2021, DALYs attributable to low whole-grain diets increased significantly, with the largest increases observed in the 50–74 years age group for both males and females (**Figure 1B**). Overall, the disease burden of CRC attributed to low whole-grain diets tends to affect younger age groups among males.

### 3.2 Regional heterogeneity in SDI-stratified burden patterns

**Figure 2:**
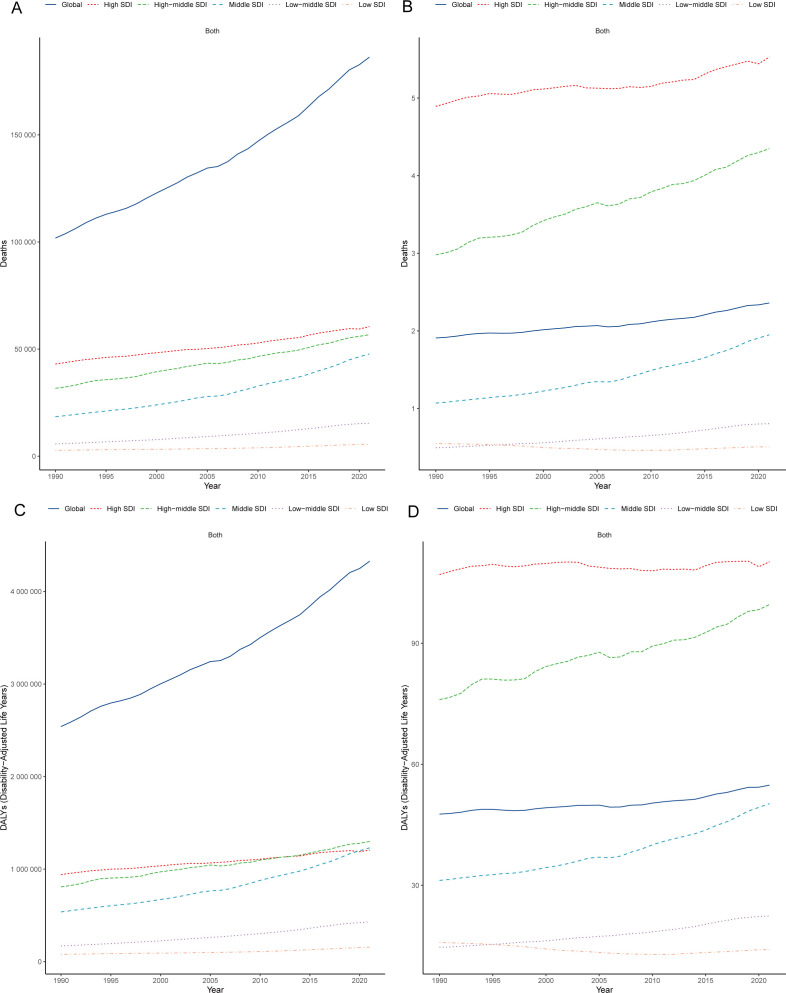
Regional heterogeneity in SDI-stratified burden patterns. Trends in CRC mortality counts **(A)** and rates **(B)** attributable to low whole-grain diets (1990–2021); Trends in CRC DALY counts **(C)** and rates **(D)** attributable to low whole-grain diets (1990–2021). CRC, colorectal cancer; DALY, disability-adjusted life years; SDI, sociodemographic index.

The burden of CRC attributable to low whole-grain diets varied across SDI quintiles. From 1990–2021, high-SDI regions (e.g., high-income Asia Pacific, high-income North America and Western Europe) consistently had the highest number of deaths and mortality rates (**Figures 2A, B**, **Table 1**). In high-SDI regions, deaths from CRC attributable to low whole-grain diets increased from 43,042.62 (95% UI: 18,344.92–64,228.50) in 1990 to 60,473.36 (95% UI: 25,199.86–92,184.17) in 2021, whereas mortality rates rose from 4.89 (95% UI: 2.09–7.30) to 5.53 (95% UI: 2.30–8.43) per 100,000 people (**Figures 2A, B**, **Table 1**). While high-SDI regions consistently bore the highest absolute burden of CRC mortality attributable to low whole-grain diets from 1990-2021, the most dramatic increases occurred in lower-SDI regions: low-middle-SDI regions experienced the largest increase in death counts (168.67%) (**Figure 2A**, **Table 1**), and middle-SDI experienced the sharpest increase in mortality rates (EAPC 1.95%) (**Figure 2B**, **Table 1**). In 2021, middle-SDI regions had the highest number of DALYs attributable to low whole-grain diets (1,230,181.64 years; 95% UI: 494,733.14–1,879,458.67), whereas high-SDI regions had the highest DALY rate (110.20 years per 100,000 people; 95% UI: 45.74–165.96) (**Figures 2C, D**; **Supplementary Table 1**). From 1990–2021, middle-SDI regions experienced the greatest increase in the DALY rate (EAPC: 1.54%; 95% CI: 1.44–1.64) (**Figures 2C, D**; **Supplementary Table 1**).

### 3.3 Disparities and trend analysis across the 21 GBD regions

**Figure 3:**
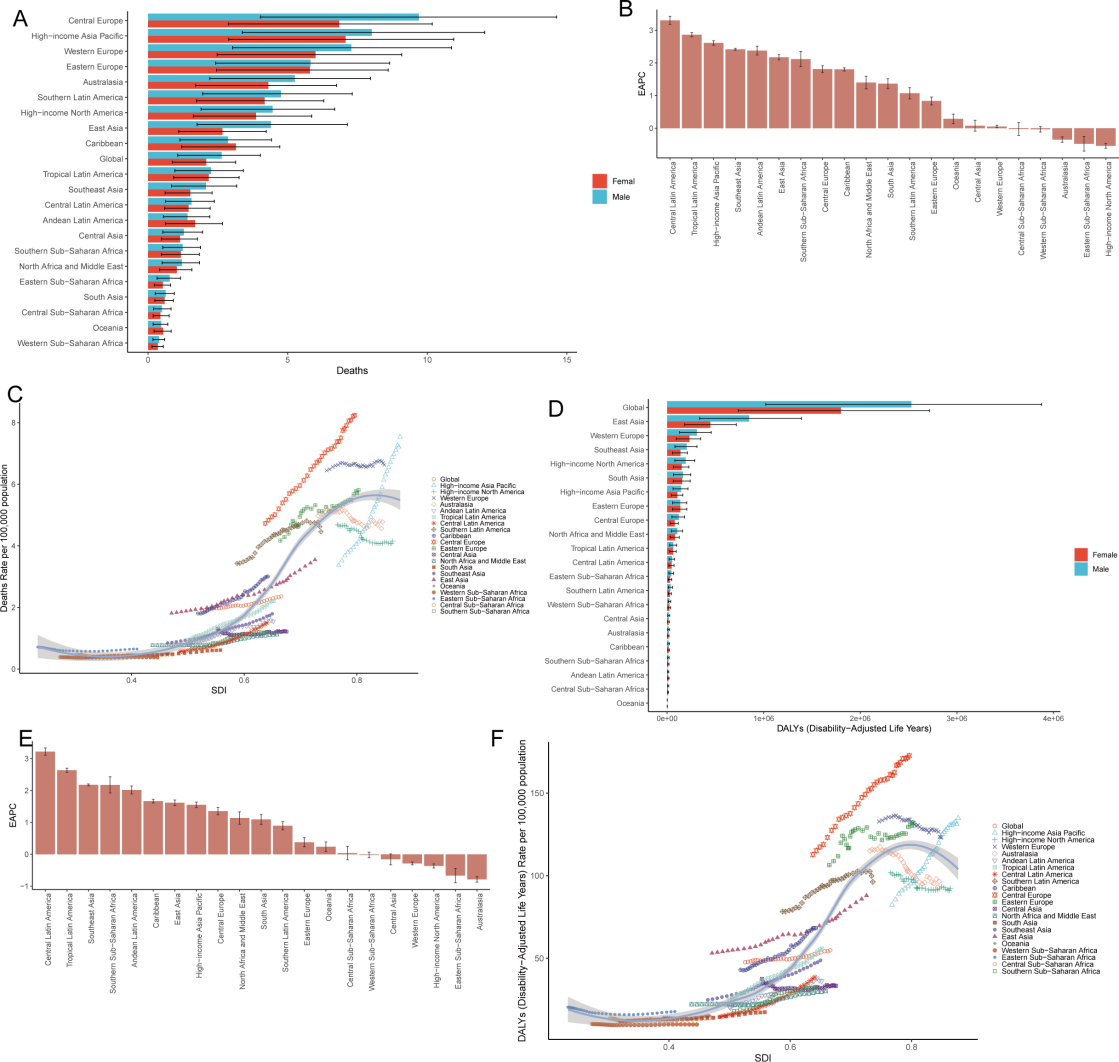
Disparities and trend analysis across the 21 GBD regions. Mortality rate **(A)** and DALYs **(D)** in 21 geographic regions in 2021; EAPC in deaths **(B)** and DALYs **(E)** in 21 geographic regions from 1990–2021; Correlation between SDI and CRC burden metrics across 21 GBD regions: mortality rates **(C)** and DALY rates **(F)**. CRC, colorectal cancer; DALY, disability-adjusted life years; SDI, sociodemographic index; EAPC, estimated annual percentage change.

The burden of CRC attributable to low whole-grain diets was compared across 21 GBD regions from 1990–2021 (**Table 1**; **Supplementary Table 1**), and the results were visualized (**Figure 3**). In 2021, Central Europe, high-income Asia Pacific, Eastern Europe, Western Europe, Australasia, southern Latin America, high-income North America, East Asia, and the Caribbean presented mortality rates that were significantly higher than the global average, with higher rates in males than in females (**Figure 3A**, **Table 1**). Central Europe had the highest mortality rate, with 18.23 deaths per 100,000 males (95% UI: 7.54–27.26) and 9.08 deaths per 100,000 females (95% UI: 3.77–13.48) (**Figure 3A**, **Table 1**). From 1990–2021, 18 regions experienced an increase in the EAPC of CRC mortality attributable to low whole-grain diets. The three regions with the greatest increases were Central Latin America (EAPC: 3.30%; 95% CI: 3.18–3.42), Tropical Latin America (EAPC: 2.86%; 95% CI: 2.80–2.93), and high-income Asia Pacific (EAPC: 2.61%; 95% CI: 2.54–2.68). High-income North America had the largest decrease in the EAPC (-0.54%; 95% CI: -0.61– -0.46) (**Figure 3B**, **Table 1**). From 1990–2021, mortality rates of CRC attributable to low whole-grain diets were positively correlated with SDI levels, with regions with higher SDIs having higher mortality rates (**Figure 3C**) (R=0.92, p<0.05).

In 2021, DALYs of CRC attributable to low whole-grain diets were generally higher in males than in females. East Asia had the highest number of DALYs (1,295,744.16 years; 95% UI: 524,659.79–2,051,943.02), followed by Western Europe (538,523.35 years; 95% UI: 222,514.13–804,551.11) and South Asia (320,917.87 years; 95% UI: 133,326.71–481,047.01) (**Figure 3D**; **Supplementary Table 1**). From 1990–2021, 16 regions experienced an increase in the EAPC for DALYs, with the largest increases in Central Latin America (EAPC: 3.22%; 95% CI: 3.11–3.34), Tropical Latin America (EAPC: 2.64%; 95% CI: 2.57–2.70), and South Asia (EAPC: 1.09%; 95% CI: 0.94–1.25); Australia had the greatest decrease in the EAPC (-0.79%; 95% CI: -0.87– -0.70) (**Figure 3E**; **Supplementary Table 1**). From 1990–2021, DALY rates were positively correlated with SDI levels, with higher SDI regions having higher DALY rates (**Figure 3F**) (R=0.91, P<0.05). In short, Central Europe presented the highest CRC mortality rates attributable to low whole-grain diets (2021), whereas Latin America experienced the most rapid increases in burden over three decades, with males consistently being more affected and higher SDI regions bearing disproportionately elevated burdens.

### 3.4 National inequalities of CRC attributable to low whole-grain diets

**Figure 4:**
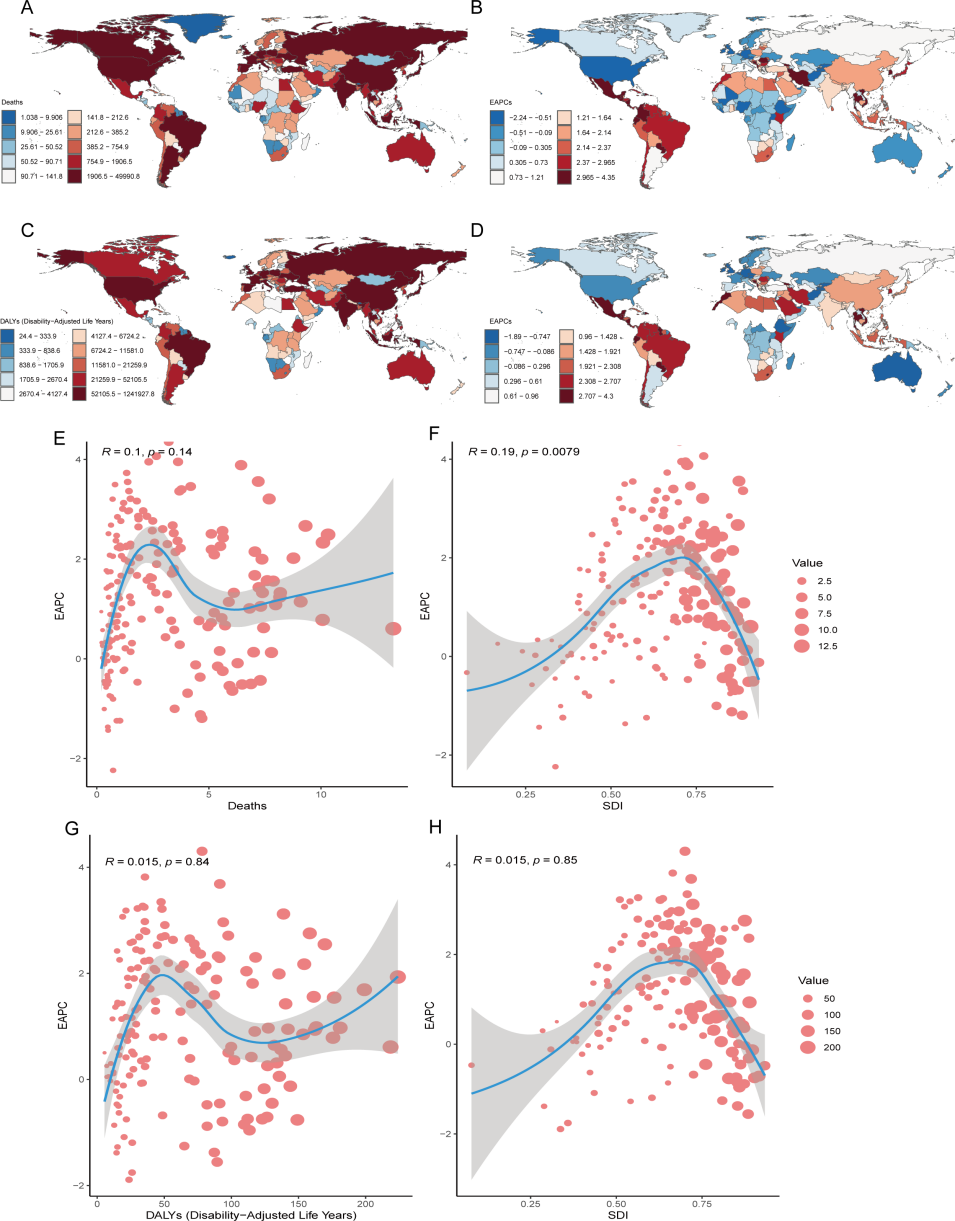
National Inequalities. National distributions of CRC burden (2021): **(A)** mortality counts, **(C)** DALY counts; Temporal trends (1990-2021): **(B)** the EAPC of age-standardized mortality rate, **(D)** the EAPC of age-standardized DALY rate; Correlation analysis of EAPC in age-standardized mortality rates with death counts **(E)** and SDI **(F)** across 204 countries; Correlation analysis of EAPC in age-standardized mortality rates with death counts **(G)** and SDI **(H)** across 204 countries. CRC, colorectal cancer; DALY, disability-adjusted life years; SDI, sociodemographic index; EAPC, estimated annual percentage change.

The burden of CRC attributable to low whole-grain diets varies by country. In 2021, Monaco had the highest mortality rate (13.23/100,000 people; 95% UI: 5.08–20.22), whereas China had the most deaths (**Figure 4A**, **Table 2**). From 1990–2021, Kuwait had the largest increase in the number of deaths (609.21%; 95% UI: 456.20–800.28), whereas Qatar and Costa Rica had the largest increases in the EAPCs for CRC mortality and rate (7.08%; 95% CI: 6.67–7.50 and 4.35%; 95% CI: 4.09–4.62, respectively) (**Figure 4B**, **Table 2**). The mortality rates and EAPCs were not significantly correlated across countries (**Figure 4E**), but the EAPC was positively correlated with the SDI (R=0.19, p<0.05) (**Figure 4F**).

**Table 2:** the top 1 nations or territories of Deaths, DALYs of CRC attributable to low whole-grain diets.

Metric (case/rate)		1990 (location, value 95% UI)		2021 (location, value 95% UI)		PC (location, value 95% UI)(%)		EAPC (location, value 95% CI)(%)	
		lowest	most	lowest	most	decrease	increase	decrease	increase
Deaths	case	Tokelau, 0.03(0.01–0.04)	China, 21,329.92(8,463.32–32,783.64)	Tokelau, 0.03(0.01–0.04)	China, 49,990.79(20,099.59–79,928.55)	Austria, -20.53(-31.26–-8.20)	Kuwait, 609.21(456.20–800.28)	Ukraine, -0.82(-0.97–-0.67)	Qatar, 7.08(6.67–7.50)
	rate	Gambia, 0.15(0.06–0.23)	Monaco,11.06(4.60–17.19)	Gambia, 0.20(0.08–0.32)	Monaco, 13.23(5.08–20.22)	Afghanistan, -51.78(-65.82–-25.71)	Mauritius, 289.96(246.22–327.35)	Afghanistan, -2.24(-2.56–-1.92)	Costa Rica, 4.35(4.09–4.62)
DALYs	case	Marshall Islands, 9.46(3.90–14.99)	China, 624,948.36(2,48199.78–959,563.32)	Marshall Islands, 21.83(9.31–36.20)	China, 1,241,927.75(50,3164.76–1,978,508.33)	Austria, -26.66(-36.11–-15.53)	Kuwait, 583.78(429.60–792.76)	Ukraine, -1.11(-1.29–-0.94)	Qatar, 7.29(6.83–7.74)
	rate	Mozambique, 5.38(2.18–8.39)	Hungary, 179.69(70.97–280.14)	Mozambique, 5.50(2.25–8.76)	Bulgaria, 224.02(86.11–347.80)	Afghanistan, -45.61(-61.43–-12.90)	Mauritius, 257.90(218.40–292.96)	Afghanistan, -1.89(-2.26–-1.52)	Costa Rica, 4.30(4.02–4.58)

CRC, colorectal cancer; DALY, disability-adjusted life years; EAPC, estimated annual percentage change; CI, confidence interval; UI, uncertainty interval.

In 2021, China recorded the highest DALY burden (1,241,927.75 years; 95% UI: 503,164.76–1,978,508.33), whereas Bulgaria presented the highest DALY rate (224.02/100,000; 95% UI: 86.11–347.80) (**Figure 4C**). From 1990–2021, Kuwait and Mauritius experienced the greatest increases in DALY counts (583.78%; 95% UI: 429.60–792.76) and rates (257.90%; 95% UI: 218.40–292.96), respectively (**Table 2**). Qatar and Costa Rica had the largest increases in EAPC for DALY number (7.29%; 95% CI: 6.83–7.74) and rates (4.30%; 95% CI: 4.02–4.58), respectively (**Figure 4D**, **Table 2**). There was no significant correlation between DALY rates and EAPC or SDI levels (**Figures 4G, H**). Globally, China and Monaco presented the highest absolute CRC burdens in 2021, whereas Kuwait presented the most explosive three-decade growth in mortality/DALY metrics, with the EAPCs in mortality rates showing a weak but significant positive correlation with national development levels (SDIs).

### 3.5 Projected burden of low whole-grain diet-attributable CRC

**Figure 5:**
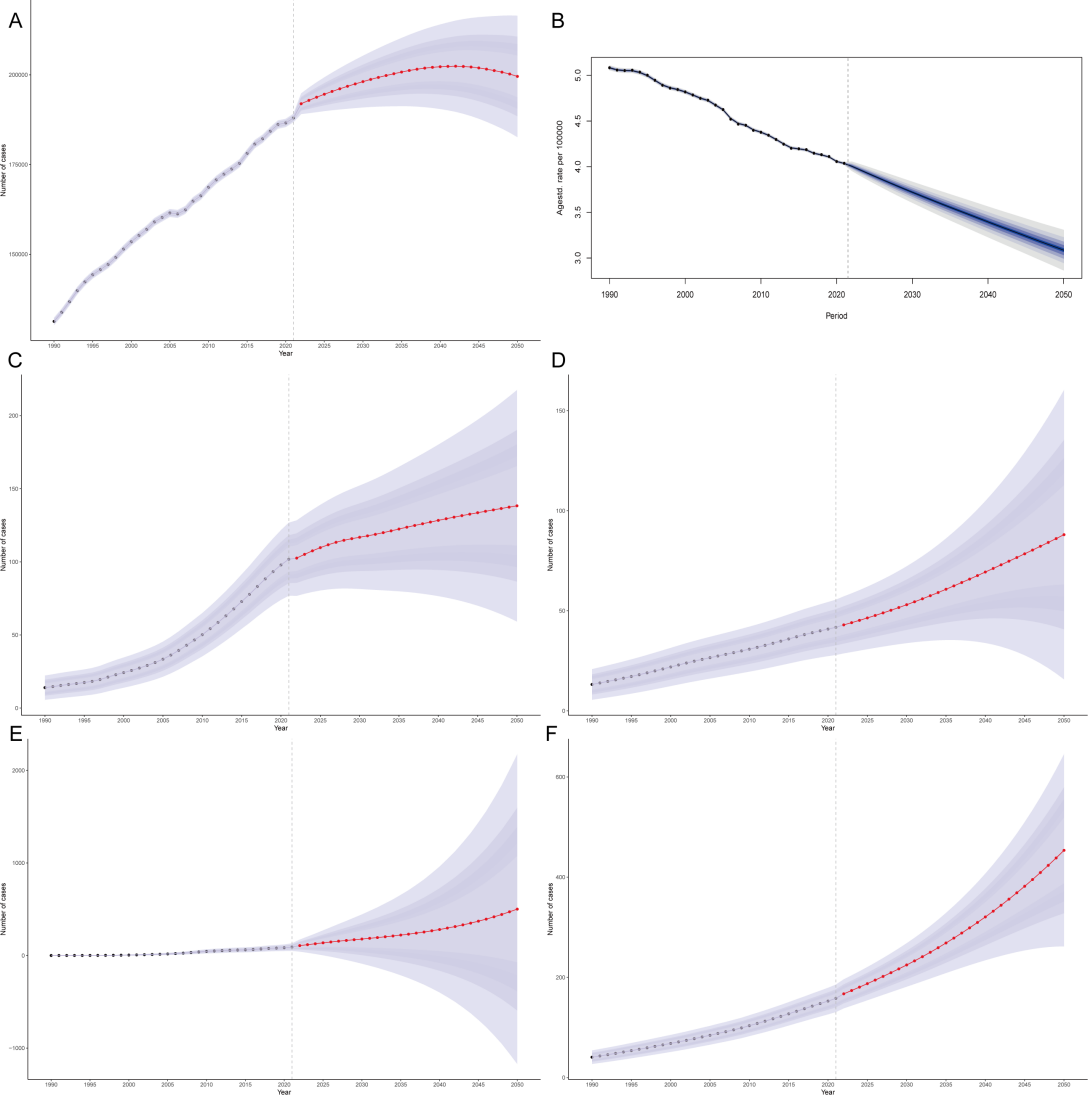
Projected burden of low whole-grain diet-attributable CRC: Global mortality counts **(A)** and rates **(B)**, with national mortality count projections for Kuwait **(C)**, Mauritius **(D)**, Qatar **(E)**, and Costa Rica **(F)**.

On the basis of the BAPC model, predictions for the period from 1990–2021 across the globe and in several countries with the most significant changes were made. The forecast indicates that from 2021–2050, the number of deaths globally due to a diet low in whole-grain-related CRC is increasing, yet the global mortality rate is declining (**Figures 5A, B**). By 2050, the global number of deaths attributable to a diet low in whole grain-related CRC is expected to increase to 199,565.06 (95% CI: 182,643.38–216,486.78), with 3.09 deaths per 100,000 people globally due to a diet low in whole grain-related CRC (**Figures 5A, B**). By 2050, the number of deaths due to a diet low in whole grain-related CRC in Kuwait, Mauritius, Qatar, and Costa Rica will reach 37,258.43 (95% CI: 30,736.18–43780.68), 88.02 (95% CI: 15.76–160.27), 501.06 (95% CI: -1,171.19–2,173.31), and 453.53 (95% CI: 261.95–645.12), respectively (**Figures 5C-F**; **Supplementary Figures 1A-D**). The predictions show that by 2050, Mauritius and Costa Rica will experience the most significant increases in mortality rates due to diets low in whole-grain-related CRC, with expected mortality rates of 10.93 and 11 deaths per 100,000 people, respectively (**Supplementary Figures 1B, D**).

## 4 Discussion

Colorectal cancer is one of the top three malignant tumors globally (1) and is a public health concern worldwide. Despite the various treatment methods available, the mortality rate remains high (1). Several dietary patterns are associated with the incidence of colorectal cancer (6, 10). A diet low in whole grains is also a contributing factor to the increased burden of CRC incidence and mortality (10). In 2019, 171,487 deaths were attributed to a diet low in whole-grain-related CRC (10). Therefore, actively reporting the burden of CRC due to low whole-grain diets can help raise public awareness about healthy eating and assist policymakers in formulating relevant policies to reduce the burden of CRC related to low whole-grain diets.

The burden of CRC related to low whole-grain diets continues to increase globally. In 2019, there were 3 million deaths worldwide due to insufficient whole grain intake (10), 171,487 of which were due to CRC (10). This study revealed that the number of deaths due to low whole-grain diets leading to CRC reached 186,256.80 in 2021 and is expected to reach 199,565.06 globally by 2050. The burden of CRC attributable to low-grain diets is further influenced by regional traditional dietary patterns globally. For example, the Asian dietary structure is characterized by refined rice as the primary staple food, compounded by excessive red meat intake, which may synergistically exacerbate CRC risk (13). While certain regions, such as China, have recognized the detrimental health impacts of low-grain diets and implemented targeted initiatives to promote public adoption of whole grains, current intake levels remain substantially below recommended thresholds (14). Conversely, traditional European diets, historically centered on meat and high animal protein consumption (15), may further contribute to increased colorectal cancer risk in the region (16). With global economic growth, the dietary structure of the public has changed. Research in China shows that Chinese residents consume more high-fat foods, excessive meat, and insufficient dietary fiber and whole grains (14). However, dietary patterns characterized by high intake of whole grains, vegetables, fruits, and dairy products and low intake of red and processed meats are associated with a lower risk of CRC (17). The intake of whole grains in Ireland is very low, with an average daily intake of 27.8 g/d, and only 19% of the population meets the recommended intake of 48 g/d (18). In the United States, the proportion of households that purchase foods containing refined grains is significantly greater than that of those that purchase foods containing whole grains (19). Whole grain intake is negatively correlated with CRC, especially rectal cancer (20). Plant-based diets, particularly those rich in whole grains, fruits, and vegetables, are associated with better survival in patients with metastatic CRC (21). Whole-grain diets can reduce systemic low-grade inflammation without causing significant changes in the gut microbiota (22). High dietary fiber in whole-grain diets may reduce the risk of CRC by increasing stool volume, thereby diluting potential carcinogens, reducing transit time through the intestines, promoting intestinal fermentation, and generating short-chain fatty acids (23). Additionally, dietary fiber can synergistically inhibit the growth of human colon cancer cells and induce apoptosis when combined with butyrate (23). Therefore, to reduce the burden of CRC due to low whole-grain diets, it is essential to strengthen education, promote whole-grain health knowledge, improve the accessibility of whole grains, widely promote the health benefits of whole grains, and encourage their consumption.

The burden of CRC due to low whole-grain diets shows sex and age trends. This study indicates that the burden of CRC due to low whole-grain diets is greater among individuals aged 50–74 years and that males are more affected than females are. The GBD 2019 study also revealed that the total burden of colorectal cancer is significantly greater in men than in women (10). Notably, this study revealed that in the 15–49-year-old age group, the CRC mortality rate due to low whole-grain diets increased significantly among men in middle–high-SDI regions (10). Men appear to be more susceptible to CRC related to dietary factors. Research by Mattiuzzi et al. (24) also revealed that the risk of colon cancer death due to the consumption of red and processed meat is greater in men than in women. KRAS gene mutations in male patients with colon cancer can inhibit ferroptosis, leading to poorer five-year survival rates (25). The sex differences in the incidence and prognosis of CRC may be attributed to differences in sex hormone levels and the gut microbiota (26). Androgens may have a direct tumor-promoting effect by increasing the proportion of opportunistic pathogens (26). Grzymislawska et al. (27) still believe that the gender trend in CRC may be related to dietary preferences. Women are more concerned about weight and consume high-fiber diets, whereas men prefer strongly flavored, greasy foods (27). Men generally consume more sodium and fewer whole grains in their diets than women do (28). In summary, these factors may exacerbate the burden of CRC and could be the cause of the sex and age trends in CRC patients due to low whole-grain diets.

The burden of CRC due to low whole-grain diets is related to the SDI. This study suggests that the higher the SDI is, the greater the burden of disease due to low whole-grain diets. With the rapid development of the food industry, people have easier access to highly processed foods, leading to a decrease in the intake of whole grains or fiber in the diet. In several economically developed regions, such as the United States, Singapore, and Denmark, the intake of whole grains is very low (29). In Singapore, 62% of children almost never consume whole-grain diets (30). This phenomenon of low whole-grain diets is particularly significant among 18–23-year-olds in the United States (31), where the intake of fruits, vegetables, and whole grains is significantly lower than that in other adults, whereas the intake of sodium, refined grains, and saturated fats is greater (31). In the United States, the highest medical costs for diet-related tumors are due to low whole-grain diets related to CRC ($2.76 billion) (32). Therefore, high-income countries especially need to focus on the burden of disease related to low whole-grain diets and take measures to reduce the burden of CRC related to low whole-grain diets to promote overall health outcomes.

Response strategies. It is widely recognized globally that whole grains are an essential part of a healthy diet, but in many countries, people still do not meet the recommended intake of whole grains. Promoting the global consumption of whole-grain diets is a primary measure to reduce the burden of CRC related to low whole-grain diets (14). First, efforts should be made to strengthen the promotion of whole-grain diets. For example, the Oldways website in the United States (wholegrainscouncil.org) offers more than 300 whole-grain recipes, free downloadable educational materials, and a database of hundreds of whole-grain health studies, which helps in promoting whole grains and expanding the consumer base (29). Special theme days can be established for promotion, such as Brown Rice Day (30) and Whole Grain Diet Day (Month) (29). Educational activities can be conducted in schools, and dietary guidelines can be provided for school cafeterias (33). Partial substitution of red meat or processed meat with plant-based foods (34). A previous study revealed that substitutions of red/processed meat with whole grains, vegetables, or fruits may substantially reduce CRC risk in high meat-consuming populations (34). Promoting the benefits of healthy diets for the public: Evidence shows that dietary modifications can extend life expectancy by 10 years (35). Second, relevant policies should be formulated, and sales channels for whole grains should be expanded. For example, the Singapore government rewards stalls that adopt whole-grain foods, funding 80% of their investment costs, while reducing the price of whole-grain diets to promote their consumption (29). Finally, for high-risk groups fed low-grain diets, early screening should be strengthened. This includes conducting fecal analysis (36), CRC mutation testing (37), colonoscopy (38), or CT scans for CRC (39, 40). In addition, health policymakers need to take more measures to strengthen education and promote whole grain health knowledge, improve the accessibility of whole grains, widely promote the health benefits of whole grains, encourage their consumption, and reduce the burden of colorectal cancer related to low whole-grain diets (14).

This study provides an in-depth analysis of the connections between the global burden of CRC and low consumption of whole grains. Globally, deaths and DALYs from low whole-grain diets causing CRC are projected to increase until 2050. There are significant differences in the burden of CRC related to low whole-grain diets across different countries and regions. Notably, in high-SDI regions, especially among men, the burden of CRC due to low whole-grain diets is the highest. Monaco has the highest mortality rate because low whole-grain diets lead to CRC, whereas China has the highest number of deaths because low whole-grain diets lead to CRC. In areas with a high incidence of CRC, in addition to improving dietary structure, more research is needed to explore the mechanisms by which whole-grain diets reduce the incidence of CRC, as well as the genetic factors of individuals. Therefore, the precise prevention and control of CRC can be achieved.

## 5 Strengths and limitations of the study

While the GBD database is extensive, it is not without its shortcomings. The GBD data differ in quality and availability depending on the region, which can result in inaccuracies, particularly in low-income nations with inadequate health systems. In many regions with well-developed health care systems, a high incidence of CRC may occur because of early screening (12). Many estimates are based on models and assumptions, potentially leading to biases. Nevertheless, the GBD continues to be an essential resource for analyzing global health patterns and guiding policy-making. The results of this study still have guiding significance for the prevention of CRC in areas with high incidence rates.

## 6 Conclusion

Low whole-grain diets pose a serious threat to public health. The discoveries made in this research provide a reference for some countries and regions globally. Health policymakers should formulate relevant policies on the basis of the current trends in the prevalence of CRC related to low whole-grain diets, strengthen the education and promotion of whole-grain diets to the public, improve their accessibility, encourage their consumption, and conduct early screening for high-risk groups to alleviate the burden of CRC related to low whole-grain diets.

## Data Availability

The original contributions presented in the study are included in the article/**Supplementary Material**. Further inquiries can be directed to the corresponding author.
