# Supplementary material for: Global burden and projection of colorectal cancer attributable to low whole-grain diets: an analysis of GBD 2021 data with Bayesian age-period-cohort modeling
Source: Front Oncol. 2025 Jul 16;15:1572053. doi: 10.3389/fonc.2025.1572053 (PMC12307195; doi:10.3389/fonc.2025.1572053)
Supplement: Supplementary file 1 [file DataSheet1.pdf]

Supplementary Figures

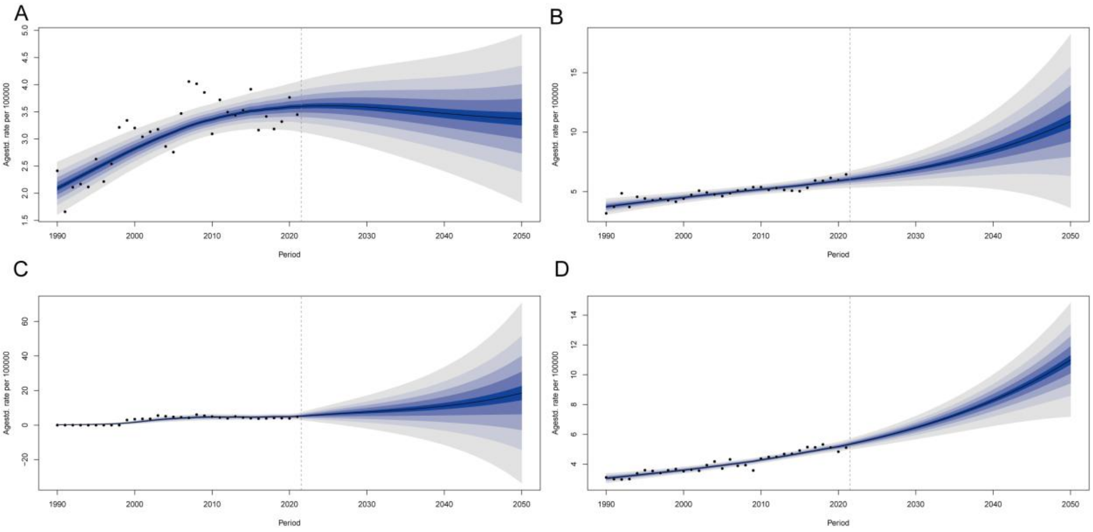

Supplementary Figure 1. Projected burden of low whole-grain diet-attributable CRC. The estimated mortality in Kuwait (A), Mauritius (B), Qatar (C), Costa Rica (D)
