# Supplementary material for: Global burden and projection of colorectal cancer attributable to low whole-grain diets: an analysis of GBD 2021 data with Bayesian age-period-cohort modeling
Source: Front Oncol. 2025 Jul 16;15:1572053. doi: 10.3389/fonc.2025.1572053 (PMC12307195; doi:10.3389/fonc.2025.1572053)
Supplement: Supplementary file 2 [file DataSheet2.pdf]

Supplementary Table 1. DALYs of CRC attributable to diet low in whole-grains between 1990–2021 at the global and regional level

Location	1990 (95% UI)		2021 (95% UI)		Case percent change % (95% UI)	EAPC Rate % (95% CI)
	case	rate	case	rate		
Global	2,540,867.41(1,050,794.36–3,754,415.59)	47.64(19.70–70.39)	4,327,218.86(1,754,865.24–6,578,232.30)	54.83(22.24–83.36)	70.30(55.66–86.21)	0.39(0.34–0.44)
High SDI	941,428.05(398,133.00–1,402,353.53)	107.04(45.27–159.44)	1,205,653.28(500,387.21–1,815,640.18)	110.20(45.74–165.96)	28.07(22.47–33.23)	0.02(-0.01–0.05)
High-middle SDI	808,497.77(332,628.85–1,194,206.54)	76.02(31.28–112.29)	1,298,819.51(519,591.61–1,965,735.77)	99.60(39.85–150.74)	60.65(44.49–82.48)	0.78(0.73–0.82)
Middle SDI	537,903.75(215,133.74–819,572.94)	31.22(12.49–47.57)	1,230,181.64(494,733.14–1,879,458.67)	50.24(20.21–76.76)	128.70(91.34–168.23)	1.54(1.44–1.64)
Low-middle SDI	170,153.86(70,941.07–262,875.67)	14.65(6.11–22.63)	430,073.38(176,445.15–643,409.64)	22.39(9.18–33.49)	152.76(117.11–209.09)	1.42(1.35–1.49)
Low SDI	79,389.16(33,441.34–123,672.58)	15.84(6.67–24.67)	157,203.40(64,961.68–233,832.04)	14.07(5.81–20.93)	98.02(68.84–173.01)	-0.55(-0.72–-0.37)
Regions						
Andean Latin America	7,809.32(3,322.01–11,899.94)	20.55(8.74–31.32)	23,978.96(9,183.44–37,638.08)	36.26(13.89–56.91)	207.06(143.15–278.06)	2.02(1.89–2.14)

Australasia	23,316.95(9,745.30–35,119.34)	115.00(48.06–173.20)	29,863.31(12,591.41–45,527.16)	96.45(40.67–147.04)	28.08(12.57–44.45)	-0.79(-0.87–-0.70)
Caribbean	15,190.28(6,477.25–22,949.97)	43.04(18.35–65.03)	32,388.67(12,893.59–49,039.06)	68.25(27.17–103.33)	113.22(84.21–144.95)	1.67(1.61–1.73)
Central Asia	25,986.97(10,919.29–39,252.40)	37.49(15.75–56.63)	31,919.29(13,173.55–48,964.30)	33.32(13.75–51.11)	22.83(7.32–38.56)	-0.16(-0.33–0.01)
Central Europe	141,071.81(58,975.30–208,941.72)	112.77(47.15–167.03)	199,028.25(82,274.51–29,7981.99)	172.67(71.38–258.52)	41.08(29.91–53.23)	1.35(1.24–1.47)
Central Latin America	24,186.86(9,945.87–35,390.33)	14.71(6.05–21.53)	97675.63(39088.78–148606.43)	38.61(15.45–58.74)	303.84(259.18–349.74)	3.22(3.11–3.34)
Central Sub-Saharan Africa	7453.95(3137.57–11526.74)	13.56(5.71–20.97)	18,960.89(7,606.00–31,746.56)	13.85(5.55–23.18)	154.37(91.70–261.19)	0.04(-0.17–0.25)
East Asia	646,779.33(257,105.92–994,559.64)	53.13(21.12–81.69)	1,295,744.16(524,659.79–2,051,943.02)	87.98(35.62–139.32)	100.34(51.66–156.40)	1.62(1.53–1.70)
Eastern Europe	240,974.85(99,959.43–357,627.47)	106.39(44.13–157.90)	273,010.20(114,998.80–401,920.89)	132.04(55.62–194.39)	13.29(3.63–23.49)	0.38(0.24–0.52)
Eastern Sub-Saharan Africa	38,963.53(16,325.85–60,926.79)	20.42(8.56–31.93)	75,702.65(31,963.04–112,168.64)	17.77(7.50–26.32)	94.29(58.27–197.26)	-0.67(-0.89–-0.44)

High-income Asia Pacific	142,754.13(59,701.38–214,811.20)	82.34(34.43–123.90)	250,020.51(105,027.07–38,0217.91)	134.82(56.63–205.03)	75.14(59.26–86.52)	1.55(1.47–1.64)
High-income North America	286,863.11(121,592.40–423,917.97)	101.94(43.21–150.64)	342,012.82(144,493.66–508,975.16)	92.39(39.03–137.50)	19.23(14.87–23.42)	-0.37(-0.43–-0.30)
North Africa and Middle East	75,303.21(31,144.43–112,922.27)	22.20(9.18–33.29)	188,025.27(75,807.81–285,507.19)	30.18(12.17–45.83)	149.69(114.10–208.74)	1.14(0.94–1.33)
Oceania	932.53(373.02–1,454.80)	14.24(5.70–22.21)	2,114.41(839.50–3,230.69)	15.18(6.03–23.20)	126.74(85.76–180.31)	0.24(0.09–0.39)
South Asia	131,068.08(56,844.82–200,092.07)	11.99(5.20–18.30)	320,917.87(133,326.71–481,047.01)	17.38(7.22–26.05)	144.85(100.17–207.82)	1.09(0.94–1.25)
Southeast Asia	115,594.06(47,680.43–177,859.55)	24.83(10.24–38.21)	340,389.08(136,101.87–511,101.24)	48.75(19.49–73.19)	194.47(147.74–248.19)	2.18(2.15–2.21)
Southern Latin America	38,792.70(16,122.62–57,863.12)	78.31(32.55–116.80)	65,166.72(26,905.01–99,425.45)	96.27(39.74–146.87)	67.99(44.98–94.54)	0.90(0.77–1.02)
Southern Sub-Saharan Africa	9,459.54(3,978.87–14,869.29)	18.05(7.59–28.37)	26,263.05(11,059.34–39,903.23)	32.70(13.77–49.69)	177.64(147.95–218.58)	2.18(1.92–2.43)
Tropical Latin America	38,033.19(15,692.38–56,901.27)	24.93(10.29–37.30)	126,985.67(54,321.21–191,290.85)	55.81(23.87–84.07)	233.88(212.40–255.63)	2.64(2.57–2.70)

Western Europe	510,577.72(215,359.73–76,5412.67)	132.82(56.02–199.11)	538,523.35(222,514.13–804,551.11)	123.12(50.87–183.94)	5.47(-1.77–11.99)	-0.29(-0.33–-0.24)
Western Sub-Saharan Africa	19,755.27(8,019.21–30,293.29)	10.23(4.15–15.68)	48,528.11(20,548.63–73,775.70)	9.91(4.20–15.06)	145.65(95.98–217.28)	-0.02(-0.11–0.07)

CRC, colorectal cancer; DALY, disability-adjusted life years; EAPC, estimated annual percentage change; CI, confidence interval;UI, uncertainty interval
